# Supplementary material for: Integrated multi-operand optical neurons for scalable and hardware-efficient deep learning
Source: Nanophotonics. 2024 Jan 8;13(12):2193–206. doi: 10.1515/nanoph-2023-0554 (PMC11501373; doi:10.1515/nanoph-2023-0554)
Supplement: Supplementary file 1 — Supplementary Material Details [file j_nanoph-2023-0554_suppl_001.docx]

Supporting Information

Integrated multi-operand optical neurons for scalable and hardware-efficient deep learning

Chenghao Feng^1,2^ , Jiaqi Gu^2,3^, Hanqing Zhu^2^, Shupeng Ning^1^, Rongxing Tang^1^, May Hlaing^2^, Jason Midkiff^2^, Sourabh Jain^1^, David Z. Pan^2^, Ray T. Chen^2,4,*^

^1^Microelectronics Research Center, The University of Texas at Austin, Austin, Texas 78758, USA.

^2^Department of Electrical and Computer Engineering, The University of Texas at Austin, Austin, Texas 78705, USA.

^3^School of Electrical, Computer and Energy Engineering, Arizona State University, Tempe, AZ 85287,

USA

^4^Omega Optics, Inc., 8500 Shoal Creek Blvd., Bldg. 4, Suite 200, Austin, TX 78757, USA.

**Supplementary Note 1: MOON training algorithms.**

To train the MOON weights while being aware of hardware noises, we adopt a hardware-aware training method where the weights are quantized mapped to the measured MOMZI responses. For each neural network layer, no matter Linear or Convolution, we map it to a general matrix multiplication operation. For each length-$k$ vector product $y=\sum_{i=1}^{k} w_{i}x_{i}$, we map $w_{i}$ and $x_{i}$ to the real chip measurement results during forward propagation,

$$w_{i}^{q}=Q_{b}\left( w_{i} \right), x_{i}^{q}=Q_{b}(x_{i})$$

$$y=f(w_{i},x_{i})$$

$y^{'}=f'\left( w_{i},x_{i} \right)=\alpha\cdot\cos\left( \sum_{i} \beta_{i}\left( Q_{b}\left( w_{i}^{q}x_{i}^{q} \right)+b_{i} \right)^{2}+\phi_{0} \right)+b$ Eq. (S1)

where $Q_{b}(\cdot)$ is the $b$-bit quantization function, $f(\cdot)$ is the look-up tanble (LUT) with 2^kb^ entries that mapps weights and inputs to the real chip measurement results, and $f'(\cdot)$ is a fitted proxy of $f\left( \cdot\right)$ that enables gradient backpropagation to $x_{i}$ and $w_{i}$. Parameters $\alpha, \beta_{i}, b_{i},\phi_{0},b$ are regressed from the curve fitting.

During backpropagation, the gradients are as follows:

$\frac{\partial L}{\partial w_{i}}=\frac{\partial L}{\partial y}\frac{\partial y^{'}}{\partial w_{i}^{q}}\frac{\partial w_{i}^{q}}{\partial w_{i}}, \frac{\partial L}{\partial x_{i}}=\frac{\partial L}{\partial y}\frac{\partial y^{'}}{\partial x_{i}^{q}}\frac{\partial x_{i}^{q}}{\partial x_{i}}, \frac{\partial w_{i}^{q}}{\partial w_{i}}=\frac{\partial x_{i}^{q}}{\partial x_{i}}=1$ Eq. (S2)

We also inject Gaussian noises $\Delta y\sim\mathcal{N}\left( 0,\sigma^{2} \right)$ to MOMZI output $(y+\Delta y)$to improve the noise robustness of the model. Output deviation $\sigma$ is input-dependent based on Fig. 5(b), i.e., $\sigma=\sigma_{0}|y|$. In this way, we fully consider physical chip responses during training to close the gap of simulation and on-chip deployment accuracy.

**Supplementary Note 2: Parameter tables for the delay, propagation loss, and footprint estimation**

**Table. S1.** Device parameters used in our performance estimation based on AIM photonics’ PDK^1^.

| Optical component | Length (μm) | Insertion loss (dB) |
| --- | --- | --- |
| High-speed EO MZI ($MZI(hs)$) | 1600 | 3 |
| High-speed plasmonic EO MZI^2^ | ~220^a^ | 11.2 |
| Low-speed TO MZI ($MZI(ls)$) | 550 | 1 |
| Microring-based filter | 16 | 0.25 |

1. Based on the layout picture of the MZI in the reference (~200 $\times100 \mu m^{2})$. The high-speed phase shifter part is only 15$\mu m$ in length, so the size of the modulator can be further optimized with more compact directional couplers.

**Table. S2.** Parameters to calculate the performance of $k$-op MOMZI-PTC.

| Parameters | Values |
| --- | --- |
| $L_{MOMZI}$ | $L_{MZI(hs)}+\left( k-1 \right)d$ |
| $L_{combiner}$ | $\frac{n}{k}L_{ring}$ |
| $d$ | 10 $\mu m$ (1.5$\mu m$ after scaling) |
| $IL_{MOON}$ | $IL_{MZI(hs)}$ |
| $IL_{combiner}$ | $\frac{n}{k}IL_{ring}$ |
| $S_{MOMZI}$ | $L_{MOMZI}\times W_{MZI}(W_{MOMZI}=460 \mu m)$ |
| $S_{combiner}$ | $\frac{mn}{k}\times L_{ring}\times W_{ring}$($W_{ring}=16 \mu m)$ |
| $S_{MZI(hs)}$ | $L_{MZI(hs)}\times W_{MZI(hs)} (MZI\left( hs \right)=460 \mu m)$ |
| $S_{MZI(ls)}$ | $L_{MZI(ls)}\times W_{MZI(ls)} (W_{MZI(ls)}=127 \mu m)$ |

**Supplementary Note 3: Energy efficiency.**

The power consumption of *n*-input, *m*-output MOMZI-PTC for computing is contributed by lasers, weight configuration, and conversion between electrons and photons, which is obtained by:

$P_{MOMZI-ONN}=P_{laser}+n(\frac{m}{k}E_{MOMZI}+E_{DAC})f_{X}+\frac{mn}{k}P_{thermal}+mP_{ADC}$ Eq. (S3)

The parameter table for modeling Eq. (S1) is provided in Table. S3. In Eq. (S3), $P_{laser}$ is the laser power. $E_{MOMZI}$ represents the energy consumption of modulators, $E_{DAC}$ and $E_{oe}$ includes the power consumption for photodetection, amplification, and analog-to-digital conversion. $f_{md}$ is the operating speed of modulators, as determined by the total delay of the MOMZI-ONN. In the MOMZI-ONN, $P_{thermal}$ is the static power to tune the MOMZI to a bias point. Using thermal phase shifters for bias tuning, $P_{thermal}=2.5 mW$. By utilizing energy-efficient active optical components based on nano-opto-electro-mechanical systems or phase change materials^3,4^, we can eliminate the power consumption for phase maintenance. To carrier-depletion-based silicon MZI modulators, $E_{MOMZI}$ can achieve ~146 fJ/bit. Using energy-efficient plasmonic-on-silicon modulators , $E_{MOMZI}=0.1$ fJ/bit. One DAC’s energy consumption can be estimated by ^5^:

$E_{DAC}=F_{D}n_{b}F_{s}/B_{r}$ Eq. (S4)

where $F_{D}$ is the DAC figure of merit, $n_{b}$ is the DAC resolution, $F_{s}$ is the sampling frequency, $B_{r}$ is the bit rate. In our estimation, $F_{D}=35$ fJ/step in a 7-nm microprocessor^6^, $n_{b}=8$ bit, $F_{s}/B_{r}=1$.

The propagation loss, the photodetectors' minimum detectable power, and the outputs' precision dictate the laser power. The total laser power can then be calculated by the following equation^7^:

$P_{laser}=m\left( \frac{n}{\rho^{2}}\frac{h\nu}{\eta IL}\max\left( 2^{2N_{b}+1},\frac{C_{d}V_{r}}{e} \right)f_{md} \right)\times\frac{n}{k}$ (Eq. S5)

where ℎ𝜈 is the photon energy at 1.55 μm, $\rho=n, \eta=0.2$ is the wall-plug efficiency of the laser^8^, $\frac{n}{k}$ is the number of wavelengths used in the MOMZI-PTC. The precision of output signals is $N_{b}$ bits. $C_{d}$ is the capacitance of the photodetector while $V_{r}$ is the operating voltage. Note that $V_{r}=0$ in some zero-biased energy-efficient photodetectors^9,10^, $f_{X}$ is the baud rate of the intput signals. In this work, we choose $f_{X}=10G$ Baud/s and use a $10$ GSPS ADC for reading the output.

**Table. S3.** Energy consumption of a *k*-point $m\times n$ MOMZI-PTC – modeling parameters

| Expression | Value |
| --- | --- |
| $E_{MOMZI}$ | ~146 fJ/bit^11^  (0.1 fJ/bit after scaling)^2^ |
| $P_{w}$ | $2.5 mW$ (PDK)  0 after scaling |
| $E_{DAC}=F_{D}N_{b}F_{s}/B_{r}$ ^5^ | $F_{D}=35$ fJ/step ^6^  $N_{b}=8 bit$  $F_{s}/B_{r}=1$ |
| $P_{laser}=\frac{mn}{k}\left( \frac{n}{\rho^{2}}\frac{h\nu}{\eta IL}\max\left( 2^{2N_{b}+1},\frac{C_{d}V_{r}}{e} \right)f_{md} \right)$ ^7^ | $\rho=n$  $\nu=193.5 THz$  $\eta=0.2$  $V_{r}=0$ |
| $P_{ADC}$(10 GSPS) | 39 mW/channel ^12^ |
|  | 0.52 fJ/level^13^ (1.3 mW/channel)  after scaling |

References

1. E. Timurdogan et al., “APSUNY Process Design Kit (PDKv3.0): O, C and L Band Silicon Photonics Component Libraries on 300mm Wafers,” 2019 Optical Fiber Communications Conference and Exhibition (OFC), 1–3, OSA (2019) [doi:10.1364/ofc.2019.tu2a.1].

2. W. Heni et al., “Plasmonic IQ modulators with attojoule per bit electrical energy consumption,” Nature Communications **10**, 1–8 (2019) [doi:10.1038/s41467-019-09724-7].

3. L. Midolo, A. Schliesser, and A. Fiore, “Nano-opto-electro-mechanical systems,” Nature Nanotechnology **13**(1), 11–18, Springer US (2018) [doi:10.1038/s41565-017-0039-1].

4. M. Wuttig, H. Bhaskaran, and T. Taubner, “Phase-change materials for non-volatile photonic applications,” Nature Photonics **11**(8), 465–476, Nature Publishing Group (2017) [doi:10.1038/nphoton.2017.126].

5. B. S. G. Pillai et al., “End-to-end energy modeling and analysis of long-haul coherent transmission systems,” Journal of Lightwave Technology **32**(18), 3093–3111, Institute of Electrical and Electronics Engineers Inc. (2014) [doi:10.1109/JLT.2014.2331086].

6. C. Huang et al., “A silicon photonic–electronic neural network for fibre nonlinearity compensation,” Nature Electronics **4**(11), 837–844, Nature Publishing Group (2021) [doi:10.1038/s41928-021-00661-2].

7. M. A. Nahmias et al., “Photonic Multiply-Accumulate Operations for Neural Networks,” IEEE Journal of Selected Topics in Quantum Electronics **26**(1), 1–18, IEEE (2020) [doi:10.1109/JSTQE.2019.2941485].

8. H. Wang et al., “High-Power Wide-Bandwidth 1.55-μm Directly Modulated DFB Lasers for Free Space Optical Communications,” in 2019 Conference on Lasers and Electro-Optics, CLEO 2019 - Proceedings, pp. JTu2A-72 (2019) [doi:10.23919/CLEO.2019.8750482].

9. L. Vivien et al., “Zero-bias 40Gbit/s germanium waveguide photodetector on silicon,” Optics Express **20**(2), 1096 (2012) [doi:10.1364/OE.20.001096].

10. T. M. Photodiodes et al., “High-Speed Evanescently-Coupled Waveguide,” 6827–6832 (2020).

11. J. Ding et al., “Ultra-low-power carrier-depletion Mach-Zehnder silicon optical modulator,” Opt. Express, OE **20**(7), 7081–7087, Optica Publishing Group (2012) [doi:10.1364/OE.20.007081].

12. “ADC (Analog-to-Digital converters) – Alphacore,” <https://www.alphacoreinc.com/adc-analog-to-digital-converters/> (accessed 30 August 2021).

13. C. Li et al., “Analog content-addressable memories with memristors,” 1, Nat Commun **11**(1), 1638, Nature Publishing Group (2020) [doi:10.1038/s41467-020-15254-4].
